# Herpes zoster mRNA vaccine induces superior vaccine immunity over licensed vaccine in mice and rhesus macaques

**DOI:** 10.1080/22221751.2024.2309985

**Published:** 2024-01-23

**Authors:** Lulu Huang, Tongyi Zhao, Weijun Zhao, Andong Shao, Huajun Zhao, Wenxuan Ma, Yingfei Gong, Xianhuan Zeng, Changzhen Weng, Lingling Bu, Zhenhua Di, Shiyu Sun, Qinsheng Dai, Minhui Sun, Limei Wang, Zhenguang Liu, Leilei Shi, Jiesen Hu, Shentong Fang, Cheng Zhang, Jian Zhang, Guan Wang, Karin Loré, Yong Yang, Ang Lin

**Affiliations:** a Vaccine Center, School of Basic Medicine and Clinical Pharmacy, China Pharmaceutical University, Nanjing, People’s Republic of China; b Center for New Drug Safety Evaluation and Research, China Pharmaceutical University, Nanjing, People’s Republic of China; c School of Life Sciences and Health Engineering, Jiangnan University, Wuxi, People’s Republic of China; d Institute of Immunopharmaceutical Sciences, School of Pharmaceutical Sciences, Shandong University, Jinan, People’s Republic of China; e Key Laboratory of Infection and Immunity, Institute of Biophysics, Chinese Academy of Sciences, Beijing, People’s Republic of China; f Targeted Discovery Center, China Pharmaceutical University, Nanjing, People’s Republic of China; g Advanced Medical Research Institute, Shandong University, Jinan, People’s Republic of China; h College of Veterinary Medicine, Nanjing Agricultural University, Nanjing, People’s Republic of China; i Precision Research Center for Refractory Diseases in Shanghai General Hospital, Shanghai Jiao Tong University School of Medicine, Shanghai, People’s Republic of China; j Firestone Biotechnologies, Shanghai, People’s Republic of China; k School of Biopharmacy, China Pharmaceutical University, Nanjing, People’s Republic of China; l Department of Immunology, College of Basic Medical Science, Dalian Medical University, Dalian, People’s Republic of China; m Department of Medicine, Solna, Karolinska Institutet and Karolinska University Hospital, Stockholm, Sweden; n School of Pharmacy, Xuzhou Medical University, Xuzhou, People’s Republic of China

**Keywords:** Varicella Zoster Virus, mRNA vaccine, Shingrix, nonhuman primate, immune mechanisms

## Abstract

Herpes zoster remains an important global health issue and mainly occurs in aged and immunocompromised individuals with an early exposure history to Varicella Zoster Virus (VZV). Although the licensed vaccine Shingrix has remarkably high efficacy, undesired reactogenicity and increasing global demand causing vaccine shortage urged the development of improved or novel VZV vaccines. In this study, we developed a novel VZV mRNA vaccine candidate (named as ZOSAL) containing sequence-optimized mRNAs encoding full-length glycoprotein E encapsulated in an ionizable lipid nanoparticle. In mice and rhesus macaques, ZOSAL demonstrated superior immunogenicity and safety in multiple aspects over Shingrix, especially in the induction of strong T-cell immunity. Transcriptomic analysis revealed that both ZOSAL and Shingrix could robustly activate innate immune compartments, especially Type-I IFN signalling and antigen processing/presentation. Multivariate correlation analysis further identified several early factors of innate compartments that can predict the magnitude of T-cell responses, which further increased our understanding of the mode of action of two different VZV vaccine modalities. Collectively, our data demonstrated the superiority of VZV mRNA vaccine over licensed subunit vaccine. The mRNA platform therefore holds prospects for further investigations in next-generation VZV vaccine development.

## Introduction

Varicella Zoster Virus (VZV) is a human alphaherpesvirus that causes varicella during primary infection and establishes latency in the sensory ganglia posing the risks for reactivation to cause herpes zoster (HZ) [1]. HZ mainly occurs in aged and immunocompromised individuals with an early exposure history and is typically symptomized by a painful dermatomal rash or more seriously postherpetic neuralgia (PHN). The genome of VZV encodes eight glycoproteins that are critical for viral entry and replication [1,2]. Amongst them, glycoprotein E (gE) is a 623-aa transmembrane protein containing a 544-aa hydrophilic ectodomain, a 17-aa hydrophobic transmembrane region, and a 62-aa cytoplasmic region. gE is abundantly present on the surface of viral particles and also on the plasma membrane of VZV-infected cells, which has been reported to mediate viral spread and skin tropism, as well as the formation of infectious virions [1]. Moreover, gE is highly immunogenic in eliciting both antibody and cell-mediated immune (CMI) responses and is believed to be a key immunogen for VZV vaccine development [2–4]. A live-attenuated vaccine (Zostavax, Merck) and a protein-based subunit vaccine containing a carboxyl-terminal truncated form of gE adjuvanted with AS01B (Shingrix) have been approved in clinical use, of which Shingrix showed a remarkably higher protective efficacy (97.2%) than Zostavax (51.3%) in adults above 50 years of age [5]. However, high reactogenicity with both local and systemic reactions and the increasing global demand causing vaccine shortage remain major concerns with Shingrix, which warrant further development of improved or novel VZV vaccines [6–9]. Recently, a domestic live-attenuated VZV vaccine developed by Changchun Bcht Biotech was approved for adult use in China, but details on the protective efficacy of this vaccine were yet undisclosed.

VZV-specific T-cell responses are critical in the prevention and control of initial VZV infection, as well as the reactivation of latent infection [10–12]. Clinical evidence has demonstrated that higher levels of VZV-specific T cells, not specific antibodies (Abs), are associated with reduced HZ incidence and disease severity [13]. More in-depth studies using simian varicella virus (SVV)-infected rhesus macaques that recapitulate VZV infection in humans showed that depletion of CD4^+^ T cells led to prolonged viremia and severe varicella during SVV infection. In contrast, the depletion of CD20^+^ B cells or CD8^+^ T cells did not alter the severity of varicella [14]. Further depletion of CD4^+^ cells in rhesus macaques with pre-established SVV latency led to viral reactivation causing zoster rash [15]. In addition, lower frequencies of VZV-specific IFN-γ and TNF-producing CD4^+^ T cells, not CD8^+^ T cells, were found to be associated with a higher incidence of HZ in patients with systemic lupus erythematosus [16]. All these suggested that there is a more central role of VZV-specific CD4^+^ T cells rather than CD8^+^ T cells or Abs in controlling VZV infection. To this end, strong virus-specific T cell responses, particularly the Th1-type CD4^+^ T cells, are indispensable to be induced by novel VZV vaccine candidates. The mRNA platform has shown multiple advantages in vaccine development including its ability to elicit strong T-cell responses, which may largely be attributed to the innate immune stimulatory effects of both mRNA cargo and lipid components in the delivery system [17–20]. A novel VZV mRNA vaccine candidate from Moderna Therapeutics has recently been tested when given as a booster dose in rhesus macaques that had been prime immunized with a live-attenuated VZV vaccine and was shown to induce comparable levels of Abs and Th1-biased T cells to Shingrix [21]. This suggested a high potentiality of mRNA technology in the development of next-generation VZV vaccines, which await further investigations.

In the current study, we developed a novel VZV mRNA vaccine (named as ZOSAL) containing sequence-optimized mRNAs encoding full-length glycoprotein E (gE) encapsulated in an ionizable lipid nanoparticle (LNP) formulation using our well-established platforms [22–24]. The immunogenicity of ZOSAL was thoroughly evaluated and compared side-by-side with the benchmark vaccine (Shingrix) in adult mice, aged mice, and importantly in rhesus macaques. To better understand the generation of vaccine-induced VZV-specific immunity and to elucidate the differences between two vaccine modalities, we performed in-depth characterization of the vaccine responses generated in rhesus macaques, with particular emphasis on the innate immune gene signatures and magnitude and functionality of B cell and T cell responses. Apart from these, some of the key safety aspects of ZOSAL and Shingrix were assessed by longitudinally monitoring the hematological and serum biochemical parameters, as well as the early changes in safety-related genes. Our data showed that, in multiple animal models, ZOSAL elicited superior VZV-specific immunity and meanwhile demonstrated higher safety profiles over Shingrix. We also identified correlates of vaccine immunity that may impact on the development of vaccine responses, which further increases our understanding of the mode of action of VZV vaccines.

## Materials and methods

A detailed description and additional materials and methods are available in supplemental materials.

## Ethics, animals, and immunization

All animal experiments were performed in accordance with the Guidelines for the Care and Use of Laboratory Animals and the Ethical Committee of China Pharmaceutical University and using protocols approved by the Institutional Animal Care and Use Committee of China Pharmaceutical University (Approval number: AP-B2209P011). C57BL/6 mice (female, 6-week or 10-month old) were purchased from Hangzhou Ziyuan Laboratory Animal Technology Co., Ltd. and randomly allocated to different groups. Mice were immunized intramuscularly (i.m.) twice at an interval of 2 weeks. Sera samples were collected longitudinally for Ab response analysis. Mice were necropsied at the indicated time points after vaccination. Spleens and draining lymph nodes (dLN) were collected and processed to obtain single-cell suspension for analysis. For the nonhuman primate (NHP) experiments, Chinese rhesus macaques (3–5 years old) were housed at the Center for New Drug Safety Evaluation and Research at China Pharmaceutical University. Animals were i.m. immunized with ZOSAL (100μg per dose) or Shingrix (human dose) at days 0 and 28. Peripheral venous blood was collected in EDTA-2 K tubes and processed within 2 h to obtain fresh peripheral blood mononuclear cells (PBMCs) according to the previously reported protocol [25]. Serum samples and PBMCs were collected longitudinally at different time points before or after vaccination. Body weight, temperature, blood cell count, and biochemical parameters were monitored longitudinally for analysis of safety profiles.

## mRNA vaccine preparation

VZV mRNA vaccine (ZOSAL) was prepared as previously described [22,23]. In brief, mRNA encoding for VZV gE immunogen was synthesized in vitro by T7 polymerase-mediated transcription from a linearized DNA template. Methyl-pseudouridine (SYNTHGENE)-modified mRNAs were capped using Cap1 Analogue Reagent (TriLink) and further purified by Monarch RNA purification columns (NEB) and resuspended in a TE buffer at the desired concentration. For mRNA encapsulation into LNP, lipid components were dissolved in ethanol at molar ratios of 50:10:38.5:1.5 (ionizable lipid: DSPC: cholesterol: PEG-lipid, all purchased from Firestone Biotechnology). The novel ionizable lipid (YX-02) was designed and patented by Firestone Biotechnologies. The lipid cocktail was mixed with mRNAs dissolved in 10 mM citrate buffer (pH4.0) at an N/P ratio of 5.3:1 and a volume ratio of 3: 1 using a microfluidic-based equipment (INano^TM^L from Micro&Nano Biologics) at a total flow rate of 12 mL/min. Formulations were diluted with PBS and ultrafiltrated using 50-kDa Amicon ultracentrifugal filters. Vaccine formulation was characterized for particle diameter, polymer dispersity index (PDI), and zeta potentials using NanoBrook Omni ZetaPlus (Brookhaven Instruments).

## Protein expression assay

Human embryonic kidney (HEK) 293T cells and DC 2.4 cells were cultured in high-glucose Dulbecco’s Modified Eagle Medium (BIOIND, Israel) supplemented with 10% fetal bovine serum (FBS, BIOIND, Israel) and 1% penicillin–streptomycin (NCM Biotech, China). gE-mRNAs were transfected into cells using jetMESSENGER (Polyplus-transfection®) and incubated for 24 h. Expression of gE protein on the cell surface was determined by staining cells with anti-gE mAb (1:200 dilution, Abcam) for 1 h prior to incubation with PE-anti-human IgG Fc (1:200 dilution, Biolgend) for 30 min. Flow cytometric analysis was carried out on Attune NxT (Thermo). Data were analyzed using FlowJo V.10.1 (Tree Star).

### Measurement of gE-specific IgG and IgG subclasses

gE proteins (Acro Biosystems) were coated into 96-well plates (Greiner Bio-One) at a concentration of 50 ng/well and incubated overnight at 4°C. The plates were washed three times with PBS containing 0.075% Tween-20 (PBST) and blocked for two hours at room temperature (RT) with 2% bovine serum albumin (BSA). Sera samples serially diluted were added and incubated for 2 h at RT. For the analysis of murine samples, binding IgG, IgG1, and IgG2c were determined using HRP-conjugated goat-anti-mouse IgG (1:50,000, Abcam), IgG1 (1:5000, Southern Biotech), IgG2c (1:5000, Southern Biotech) Ab for 1 h at 30°C, respectively. For the analysis of the NHP sample, binding IgG was determined using HRP-conjugated goat anti-monkey IgG (1:50,000, Abcam) for 1 h at 30°C. TMB substrate was used for development and the absorbance was read at 450 nm wavelength. The endpoint titre was calculated as the dilution factor that emitted an optical density (OD) value above 4.1×background.

### Analysis of memory B cell response

Frequencies of VZV gE-specific class-switched IgD^-^IgM^-^ MBCs were assessed by flow cytometry. For the preparation of gE-probes, biotinylated gE proteins (Acro Biosystems) were conjugated with BV421- or APC-streptavidin (Biolegend) at a molar ratio of 4:1. Cells from mouse spleen or dLN or PBMC from rhesus macaques were first incubated with gE-probes for 20 min, and then stained with Fixable Viability Dye eFluorTM 506 (eBioscience) for 5 min. After washing, cells were incubated with Fc Receptor blocking reagent (Miltenyi) and antibody cocktails for 20 min at 4°C in the dark. Flow cytometric analysis was carried out on BD FACSymphony A3 (BD Biosciences). Data were analyzed using FlowJo V.10.1 (Tree Star). A list of antibodies used in this analysis is available in supplemental materials.

### Antigen recall T cell assay

For the evaluation of gE-specific T cell response, a total of 2 million murine splenocytes or rhesus PBMCs were seeded per well into 96-well U-bottom plates and incubated with or without gE protein (2 μg/mL, Acro Biosystems) or gE overlapping peptides (10 μg/mL, 153 peptides, 15mers with 11 aa overlap) in the presence of brefeldin A (BFA, Biolegend) for 8 h or 16 h at 37°C, respectively. Cytokine production of T cells was evaluated by surface and intracellular staining using the Fixation/Permeabilization Solution Kit (BD Biosciences). Background cytokine staining was subtracted, as defined by staining in the samples incubated with medium alone. A list of antibodies used in this analysis is available in the Supplemental Tables 1 and 2.

### Enzyme-linked immunospot (ELISPOT) assay

Mouse splenocytes or rhesus macaques PBMCs (0.2 million cells per well) were incubated with or without gE protein (1 μg/mL, Acro Biosystems) for 20 h. Frequencies of IFN-γ or IL-2-secreting T cells were assessed using commercial kits (Mabtech) according to the manuals. Spots were developed with BCIP/NBT substrate (Mabtech) and counted using CTL-Immunospot S6 Analyzer. Results were depicted as spot-forming cell (SFC) per million stimulated cells.

### Statistical analysis

Statistical calculations were performed using GraphPad Prism v6.0. Comparisons between groups were determined using two-way analysis of variance (ANOVA) or Mann–Whitney U test. Comparisons between two different time points within the same vaccine group were analyzed using 2-tailed paired Wilcoxon’s test. A *p* value less than 0.05 was considered statistically significant (*p ≤ 0.05, **p ≤ 0.01, ***p ≤ 0.001, ****p ≤ 0.0001).

## Results

### Rational design and characterization of a novel VZV mRNA vaccine candidate (ZOSAL)

The glycoprotein E (gE) is a dominant immunogen of VZV and has been widely used as a key antigen candidate for VZV vaccine development. Different from Moderna’s VZV mRNA vaccine encoding for a carboxyl-terminal truncated form of gE antigen [21], we designed our mRNA vaccine encoding the full-length gE antigen considering that efficient T cell epitopes were reported to span the whole protein [26]. Recently, we reported a proprietary artificial intelligence (AI)-based algorithm (LinearDesign) that can design mRNA sequence to achieve optimal folding stability and codon usage that together contribute to high translation efficiency and high vaccine immunogenicity [24]. Using this AI tool, we designed a codon-optimized gE-mRNA sequence based on the wide type sequence from the VZV Oka strain (GenBank: AY253715.1). The optimized gE-mRNAs were synthesized using in-vitro transcription (IVT) procedures with N1-methyl-pseudouridine (m1Ψ) modification and showed robust and efficient protein expression upon transfection into HEK-293T and DC2.4 cells (Figure S1a, b). VZV mRNA vaccine formulation (named as ZOSAL) was further prepared by encapsulating gE-mRNAs into the LNP delivery system using a microfluidics-based procedure. Transmission electron microscopy and dynamic light-scattering analyses confirmed a high homogeneity in both shape and size of the nanoparticle, which demonstrated a particle size of 105.3 ± 1.6 nm with a polymer dispersity index (PDI) below 0.1 analyzed by Dynamic light scattering (Figure S1c, d).

### ZOSAL induced robust VZV-specific Ab and memory B cell responses in mice

**Figure 1.**
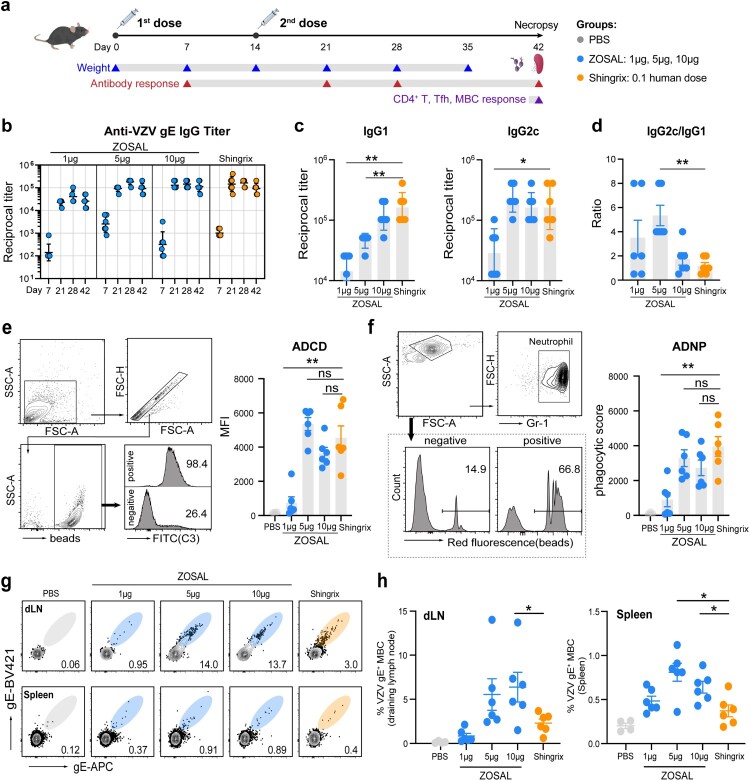
Antibody and memory B cell responses induced by ZOSAL and Shingrix in mice. a. Experimental design. C57BL/6 mice (*n* = 6) were i.m. immunized with escalating doses of ZOSAL or 0.1 human dose of Shingrix on day 0 and day 14. Blood draws were taken at the indicated time points. Spleens and dLNs were collected 28 days after the boost. b. Anti-gE IgG titres were measured by ELISA and endpoint titres are shown. **c.** Anti-gE IgG1 and IgG2c titres at day 28 were measured by ELISA and endpoint titres are shown. d. Ratio of IgG2c/IgG1 is shown. e-f. ADCD and ADNP functions of Abs were analyzed using sera collected on day 28. gE-coated microbeads were incubated with diluted and heat-inactivated sera. ADCD (e) was detected by fluorescently labelled anti-C3 Abs and MFIs are shown. ADNP (f) was determined by beads-positive primary neutrophils and phagocytic scores are shown. g-h. Frequencies of class-switched (IgD^-^IgM^-^) gE-specific MBCs in dLNs and spleens were assessed by flow cytometry. Data are shown as mean ± SEM. Mann-Whitney U test was used for statistical analysis. *p ≤ 0.05, **p ≤ 0.01.

Immunogenicity of ZOSAL and Shingrix was first evaluated and compared in C57BL/6 mice that were i.m. immunized with escalating doses (1, 5, and 10 μg) of ZOSAL or 0.1 human dose of Shingrix on day 0 and day 14 (Figure 1a). Following vaccination, we noticed a transient loss of body weight 7 days after booster in mice from the Shingrix group (Figure S2), which supported high reactogenicity of Shingrix in line with clinical observation [6,7]. In contrast, ZOSAL-vaccinated mice showed a steady increase in body weight indicating a good safety profile. With regards to the Ab response, two doses of ZOSAL induced robust levels of gE-specific IgG in a dose-dependent manner. The response was more prominent in mice receiving medium (5 μg) or high dose (10 μg) mRNA vaccine and was at a comparable level to that induced by Shingrix (Figure 1b). Further by analyzing gE-specific IgG subclasses, we found that ZOSAL was more potent at inducing Th1-prone IgG responses that are demonstrated by induction of a lower IgG1 titre and higher IgG2c/IgG1 ratio (Figure 1c, d). Fc-mediated Ab functions, including Ab-dependent complement deposition (ADCD) and Ab-dependent neutrophil phagocytosis (ADNP), were also assessed by systems serology approaches using sera collected 14 days after the boost [27]. Despite a distinct IgG subclass composition observed (Figure 1d), there was no difference in ADCD (Figure 1e) and ADNP (Figure 1f) effects between the two vaccine groups. Since Ab function is affected not only by the subclass but also by the patterns of glycosylation of Fc region. Whether ZOSAL and Shingrix induce different glycosylation patterns of Abs awaits further investigation. In addition, we measured the frequencies of class-switched IgD^-^IgM^-^ memory B cells (MBCs) specific to gE antigen in spleens and lymph nodes draining the vaccine injection site (*d*LN) 4 weeks after the boost. Two doses of 5 μg or 10 μg ZOSAL elicited a significantly higher level of gE^+^ MBCs than Shingrix, which was found in both two lymphoid organs dissected (Figure 1g, h). This suggested that ZOSAL was likely to induce a more sustained vaccine response than Shingrix.

### ZOSAL induced stronger gE-specific T cell responses than Shingrix in mice

**Figure 2.**
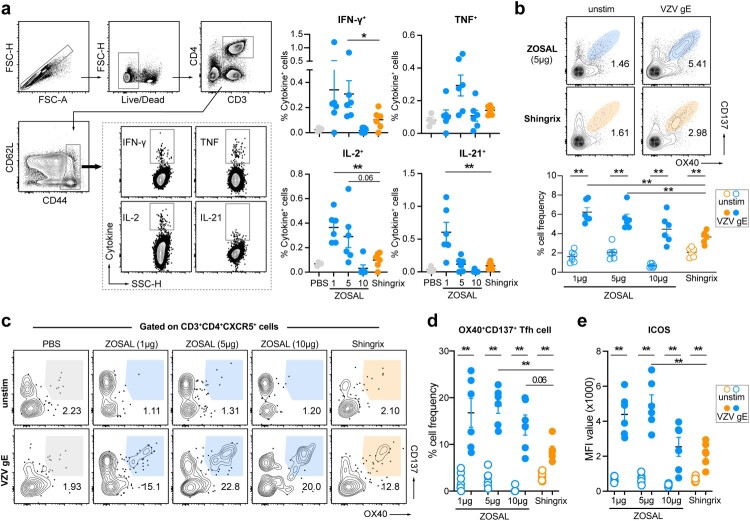
T cell responses induced by ZOSAL and Shingrix in mice. C57BL/6 mice (n = 6) were i.m. immunized with escalating doses of ZOSAL or 0.1 human dose of Shingrix on day 0 and day 14. Spleens were collected 28 days after the boost immunization. a. Splenocytes were stimulated with or without gE antigen (2 μg/ml) for 8 h in the presence of Brefeldin A. Frequencies of IFN-γ, IL-2, TNF, or IL-21-secreting CD4^+^ T cells were analyzed by flow cytometry. Data from one representative animal is shown. b–d. Splenocytes were stimulated with or without gE antigen (2 μg/ml) for 20 h. Frequencies of AIM ^+ ^CD4^+^ T cells (b) and AIM+ Tfh cells (c,d) were determined by flow cytometry. e. Expression of ICOS on OX40 ^+ ^CD137 ^+ ^CXCR5 ^+ ^CD4^+^ Tfh cells was evaluated. MFI value is shown. Data represent mean ± SEM. Mann-Whitney U test was used for statistical analysis. *p ≤ 0.05, **p ≤ 0.01.

As Th1-biased CD4^+^ T cell response is critical for the prevention and control of VZV [16,28], we used an intracellular cytokine recall assay to evaluate the induction of gE-specific CD4^+^ T cells and their production of IFN-γ, TNF, IL-2, as well as IL-21 at day 28 after boost immunization. Compared with Shingrix, ZOSAL vaccination elicited significantly higher frequencies of Th1-type CD4^+^ T cells in spleens. Interestingly, the induced T cell response was more prominent in mice receiving low or medium doses of mRNA vaccine (Figure 2a), which did not agree with the dose-dependent increasing trend observed in Ab responses described above (Figure 1b). Moreover, we detected a stronger induction of gE-specific AIM^+^ (activation-induced marker, OX40 and CD137) CD4^+^ T cells upon antigen stimulation in the spleens of ZOSAL-vaccinated mice (Figure 2b). Frequencies of cytokine-producing CD8^+^ T cells were also assessed by FACS but showed no clear induction by the two vaccines (data now shown). T follicular helper (Tfh) cells that are specialized in providing helper signals to B cells and are critical for germinal centre reaction were also measured in parallel in our study. We found that gE-specific Tfh cells defined as OX40 ^+ ^CD137 ^+ ^CD4 ^+ ^CXCR5^+^ T cells were significantly expanded in spleens, especially in mice vaccinated with ZOSAL (Figure 2c, d). Moreover, these vaccine-induced Tfh cells showed a clearly activated phenotype indicated by an elevated expression of ICOS (Figure 2e). All these together showed that ZOSAL was more potent at inducing VZV-specific T-cell responses than Shingrix in mice.

As the incidence of herpes zoster increases with age [8], we also evaluated and compared the immune responses induced by ZOSAL and Shingrix in aged C57BL/6 mice (10-month old) following two doses of vaccination at day 0 and day 14, respectively. While anti-gE IgG titres were at comparable levels between two vaccine groups (Figure S3a), aged mice immunized with ZOSAL demonstrated a lower level of IgG1, a higher level of IgG2c, and a remarkably higher IgG2c/IgG1 ratio (Figure S3b, c). Regarding gE-specific T cell responses, ZOSAL induced a significantly higher level of IFN-γ or IL-2-producing T cells than Shingrix (Figure S3d, e). These data were in line with the above findings in adult mice and further supported the superior immunogenicity of ZOSAL.

### Innate immune responses induced by ZOSAL and Shingrix in mice and rhesus macaques

Sufficient stimulation of innate immunity by vaccination is fundamental to the generation of high-quality vaccine-specific adaptive responses. To this end, we next studied the innate immune responses in C57BL/6 mice that were i.m. administered with different doses of ZOSAL (1, 5, and 10 μg) or corresponding equivalent amount of empty LNP, which were benchmarked to Shingrix. We found that ZOSAL induced significant dose-dependent activation of two major dendritic cell (DC) subsets, cDC1 and cDC2 both in the spleens and dLNs at an early time point (12 h) after vaccine administration (Figure S4a-b). Shingrix induced strong activation of DCs in the dLNs but not in the spleens (Figure S4b). In addition, an empty LNP vector also induced splenic cDC1 and cDC2 activation, albeit at a much lower level than LNP containing mRNA (ZOSAL), which suggested an immune-stimulatory effect of LNP as previously reported [29,30]. Serum alanine aminotransferase (ALT) and aspartate aminotransferase (AST) levels were also assessed, which were not altered early after vaccination and therefore suggested there was no acute liver toxicity induced (Figure S4c).

**Figure 3.**
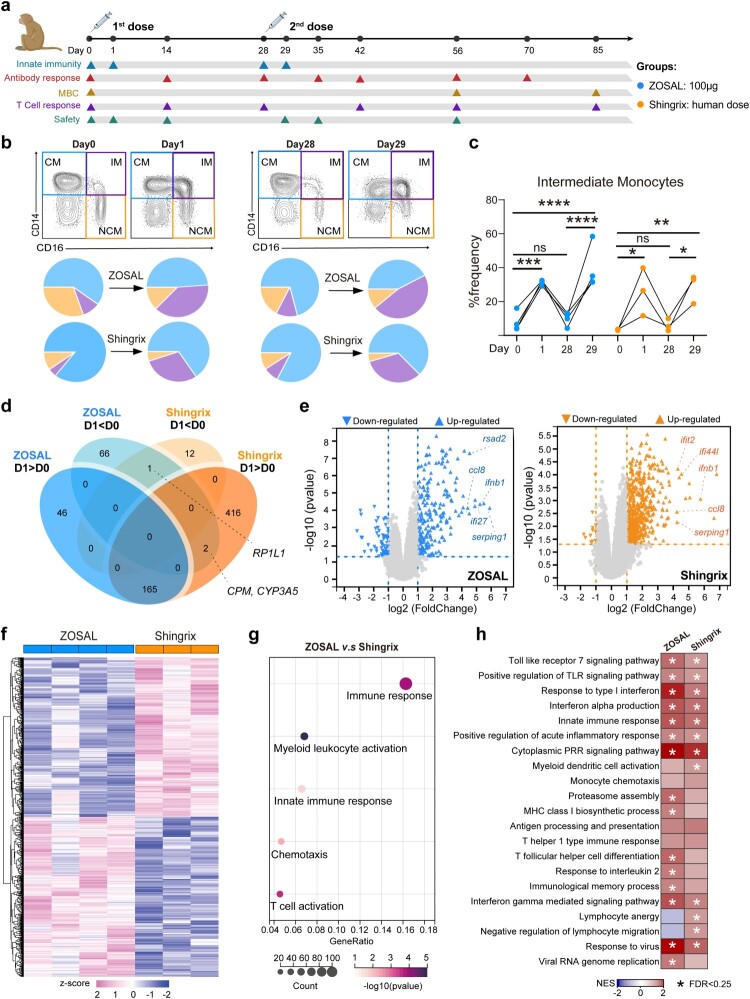
Alteration of intermediate monocytes and modulation of gene expression after ZOSAL and Shingrix vaccination in rhesus macaques. a. Experimental design. Rhesus macaques were i.m. immunized twice with ZOSAL (*n* = 4) or Shingrix (*n* = 3) at an interval of 4 weeks. Blood and serum samples were taken at the indicated time points for analysis. b. Differentiation of three monocyte subsets 24 h after prime and boost immunization is shown. Classical monocyte (CM), intermediate monocyte (IM), or nonclassical monocyte (NCM). Pie charts indicate the percentage of each monocyte subset out of total monocytes. c. Frequencies of CD14 ^+ ^CD16^+^ intermediate monocytes are shown. d-h. Transcriptomic analyses of PBMCs isolated 24 h post the boost immunization. d. The Venn diagram indicates the number of altered genes shared by the two vaccine groups. D1 > D0 and D1 < D0 represent up-regulated and down-regulated genes, respectively. e. Volcano plots display genes that were altered by ZOSAL or Shingrix vaccination. Criteria used are *p* value < 0.05 calculated using a paired two-tailed Student’s *t* test and at least 2-folded change after vaccination. Representative genes are annotated. f. Heatmaps of significantly altered DEGs by ZOSAL or Shingrix are shown. Values in heatmaps are z-score standardized. g. DEGs in ZOSAL-vaccinated animals versus Shingrix-vaccinated animals were employed for GO analysis, with a focus on the indicated GO biological process terms. h. GSEA analysis using all DEGs. Each box represents a specific module and colours indicate normalized enrichment score (NES). The asterisk denoted in the box represents False Discovery Rate (FDR) values < 0.25. Two-way ANOVA was used for statistical analysis in figure c. *p ≤ 0.05, **p ≤ 0.01, ***p ≤ 0.001, ****p ≤ 0.0001.

Nonhuman primate serves as an important animal model for preclinical testing of vaccines due to their genetic and physiological similarities to humans. To better assess our mRNA vaccine and gain mechanistic insights into the generation of VZV immunity by two different vaccine modalities, we next performed an in-depth characterization of the vaccine responses induced by ZOSAL and Shingrix in rhesus macaques (Figure 3a). Two doses of ZOSAL (100 μg) or Shingrix (human dose) were i.m. administered at an interval of 4 weeks. We first measured the early innate immune responses after vaccine administration by monitoring the fluctuation of distinct leukocyte subsets. 24 h after prime immunization, both ZOSAL and Shingrix were shown to induce a rapid and transient decrease in circulating lymphocytes, accompanied by a transient increase in neutrophils and monocytes (Figure S5). Among the expanded monocytes, there was a noticeable increase of CD14 ^+ ^CD16^+^ intermediate monocytes not only in cell frequency but as a proportion within the monocyte compartment (Figure 3b, c), which was slightly more pronounced in the ZOSAL vaccine group and was found both after prime and booster dose. These findings coincided with what we have previously reported for several other vaccines including a COVID-19 mRNA vaccine [31], a rabies mRNA vaccine [32], and a protein-based subunit malaria vaccine [33], which reflect a strong innate immune activation.

To gain a more in-depth understanding of the innate immune activation, we performed transcriptomic analyses on PBMCs isolated 24 h post the boost immunization. Profiles and changes in gene expression were thoroughly evaluated using bioinformatic approaches. After vaccination, there was a considerable overlap of 165 genes showing ≥ 2-fold increase and only one gene (*RP1L1*) showing ≤ 0.5-fold decrease that was shared by the two vaccine groups when compared with matched pre-vaccination (day 0) samples (Figure 3d). Two other genes, namely *CPM* and *CYP3A5* were down-regulated after ZOSAL vaccination but, in contrast, were increased upon Shingrix vaccination. The exact physiological outcomes of changes in *CYP3A5* expression by two different vaccines remain unknown, but it was likely to be associated with drug metabolization and the resulting toxicity since *CYP3A5*-encoded liver enzyme is critical to metabolize and detoxify xenobiotics [34,35]. Genes that were altered in their expression by the two vaccines were further identified, amongst which a large number of genes (*ccl8, ifnb1, serping 1*, etc) related to interferon (IFN) response, complement pathway, and chemotaxis were up-regulated and commonly shared by the two groups (Figure 3e). And, it was clearly observed that ZOSAL and Shingrix showed distinct impact on gene expression patterns (Figure 3f). Further, differentially expressed genes (DEGs) were subjected to a Gene Ontology (GO) analysis using the GO biological process database and were found to be highly enriched in pathways related to “immune response,” “myeloid leukocyte activation,” “innate immune response,” “chemotaxis” and “T cell activation” (Figure 3g). These DEGs were depicted in Supplemental Table 3. Furthermore, Gene Set Enrichment Analysis (GSEA) was performed for in-detail characterization of their functions and enrichments in gene modules. Modules including TLR signalling, Type-I IFN response, cell chemotaxis, antigen processing and presentation, lymphocyte function, etc that are closely related to the generation of vaccine responses were in general highly upregulated in both two groups (Figure 3h, Table S4), which was more prominent in ZOSAL-vaccinated rhesus macaques. Interestingly, although Shingrix is potent at inducing strong T cell responses, genes associated with “lymphocyte anergy” and “negative regulation of lymphocyte migration” concomitantly increased. In contrast, these two modules were clearly decreased upon ZOSAL vaccination (Figure 3h). This further suggested the superiority of ZOSAL over Shingrix in eliciting stronger T cell immunity in rhesus macaques as we studied later (Figure 5).

### ZOSAL induced comparable levels of Ab and MBC responses to Shingrix in rhesus macaques

**Figure 4.**
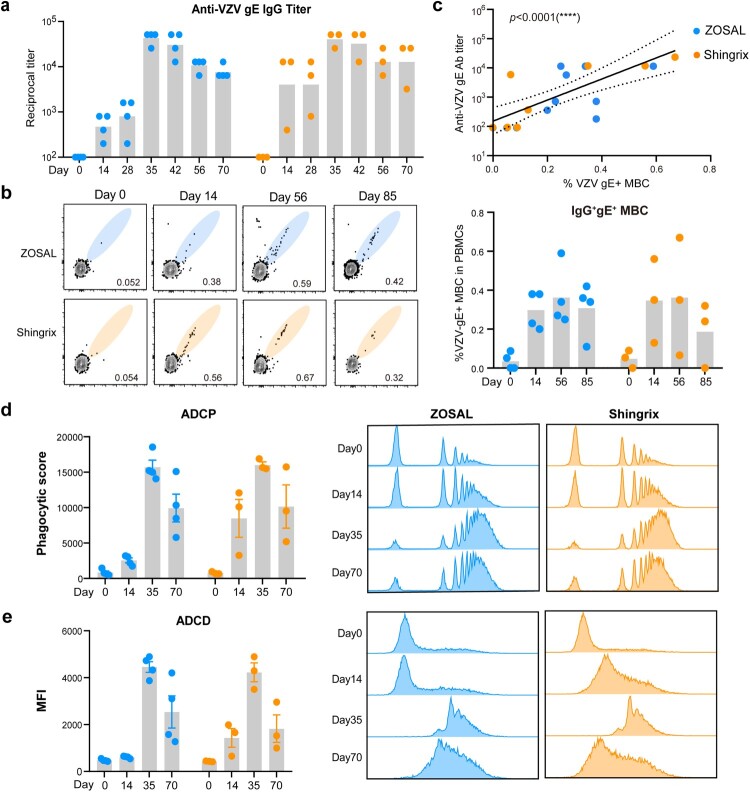
Antibody and memory B cell responses induced by ZOSAL and Shingrix in rhesus macaques. Rhesus macaques were i.m. immunized twice with ZOSAL (n = 4) or Shingrix (n = 3) at an interval of 4 weeks. a. Endpoint titres of anti-gE IgG were measured by ELISA longitudinally. b. Frequencies of class-switched IgD^-^IgM^-^ gE-specific MBCs in PBMCs were assessed by flow cytometry. Data from representative animals (left panel) and cell frequencies are shown as mean ± SEM (right panel). c. Correlation of class-switched gE^+^ MBCs and anti-gE IgG titres. d. gE-coated microbeads were incubated with diluted and heat-inactivated sera, followed by incubation with THP-1 cells. ADCP effect of Abs was determined as frequencies of beads-positive cells and phagocytic scores are shown. e. ADCD effect of Abs was detected by fluorescently labelled anti-C3 Abs and MFIs are shown. Pearson’s correlation analysis was used. Data are shown as mean ± SEM. ****p ≤ 0.0001.

We next evaluated the VZV-specific Ab and B cell responses induced by ZOSAL and Shingrix in rhesus macaques. Consistent with what we have found earlier in the mouse model (Figure 1), both two vaccines were able to induce detectable levels of anti-gE IgG after prime immunization, and the titres increased remarkably reaching a comparable and peak level 7 days after the boost (Figure 4a). Class-switched gE-specific MBCs in PBMCs were also analyzed according to the indicated gating strategy (Figure S6a), which were robustly induced by both two vaccines at an equivalent level and their cell frequencies correlated well with Ab titres (Figure 4b, c). Functions of Abs including Ab-dependent cellular phagocytosis (ADCP) by THP-1 cells and ADCD effects were also evaluated using sera collected at different time points after vaccination. No clear difference was observed regarding the functions of Abs induced by ZOSAL and Shingrix, which was in line with our findings in C57BL/6 mice (Figure 1e, f).

### ZOSAL was more potent at inducing a Th1-biased CD4^+^ T cell response than Shingrix in rhesus macaques

**Figure 5.**
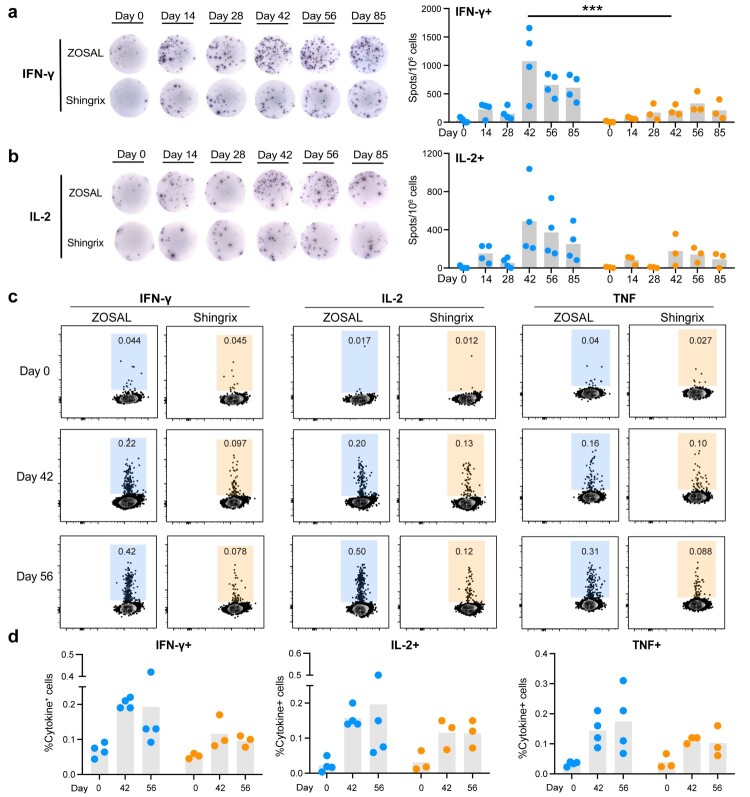
A stronger Th1-biased CD4+ T cell response was induced by ZOSAL than Shingrix in rhesus macaques. Rhesus macaques were i.m. immunized twice with ZOSAL (n = 4) or Shingrix (n = 3) at an interval of 4 weeks. a-b. PBMCs collected at different time points before and after vaccination were stimulated with gE antigen (2 μg/ml) for 20 h. Frequencies of IFN-γ or IL-2-secreting T cells at the indicated time points were measured by ELISpot. Data from representative animals are shown. Numbers of gE-specific cytokine-producing T cells were enumerated and are shown as spots per million stimulated cells. c. PBMCs were stimulated with or without gE overlapping peptides pool (10 μg/ml) for 16 h in the presence of Brefeldin A. Frequencies of IFN-γ, IL-2, TNF-secreting CD4^+^ T cells were analyzed by flow cytometry. Data from representative animals are shown. d. Quantification of cytokine-producing CD4^+^ T cells upon antigen stimulation. Two-way ANOVA with multiple comparison tests was used for analysis of statistical significance. ***p ≤ 0.001.

Given that Th1-type T cells, not Abs, mediate the protection against VZV [10–12], we gave more attention to the T cell responses elicited. Using ELISpot assays, we found that a single prime dose of ZOSAL could induce a moderate level of IFN-γ or IL-2 secreting T cells at a higher magnitude than Shingrix (Figure 5a, b). Such response was markedly enhanced following the boost immunization, especially in animals receiving ZOSAL vaccination. As previous study has validated a more critical role of CD4^+^ T cells rather than CD8^+^ T cells in protection against varicella virus [14,16], we further used a well-established intracellular cytokine recall assay (Figure S6b**)** to evaluate the induction of gE-specific CD4^+^ T cells producing Th1-type cytokines (IFN-γ, TNF, IL-2) upon stimulation by gE overlapping peptides pool. Two or four weeks after the boost immunization, ZOSAL vaccination elicited significantly higher frequencies of Th1-type CD4^+^ T cells than Shingrix, although no statistical significance was observed due to the limited number of animals used. Frequencies of cytokine-producing CD8^+^ T cells were also analyzed in parallel, which showed no clear induction in both two vaccine groups (Figure S7). Combined with the findings in a mouse model (Figure 2), these results collectively showed that ZOSAL was superior at inducing VZV-specific CD4^+^ T cell immunity than Shingrix.

### Correlation matrix and cluster analysis of immune parameters identified

**Figure 6.**
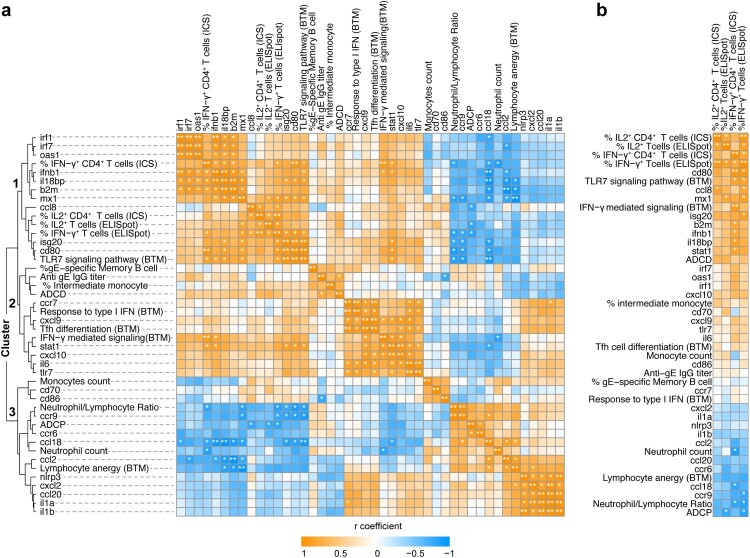
Correlation and cluster analysis of immune parameters. a. A multivariate nonparametric Spearman’s test was used to analyze the correlation among 44 immune parameters measured in the study. The heatmap shows the correlation coefficient with four clusters denoted. *p ≤ 0.05, **p ≤ 0.01. b. Immune parameters correlating with gE-specific Th1 T cell responses were isolated and shown.

Longitudinal sample collection and the above comprehensive analyses gave us an opportunity to elucidate the correlations among key immune parameters identified, which help us better understand the generation of vaccine-induced VZV immunity. We therefore took the step further to perform a multivariate nonparametric correlation analysis using 44 variables measured in our study followed by hierarchical clustering as described previously [33]. Early innate immune parameters correlating with adaptive vaccine responses were particularly emphasized. Three clusters were identified in our analysis (Figure 6a). Amongst them, cluster 1 that contains genes relevant to IFN response (*mx1, isg20*), antigen presentation (*b2m, cd80*), and TLR7 signalling pathway showed highly positive correlations with gE-specific Th1-type T cell responses. Interestingly, cluster 3 that includes neutrophil count and neutrophil/lymphocyte ratio measured at day 1, ADCP function of Abs at day 35, etc showed negative correlations with cluster 1 representing T cell responses. The most critical endpoints in our study, that is frequencies of Th1-type T cells, were further isolated for a better visualization of their associating variables. It was clearly demonstrated that vaccine-induced T-cell responses were highly and positively correlated with the expression of IFN-stimulated genes. While genes related to chemotaxis (ccr6, ccr9), ADCP function of Abs, and neutrophil/lymphocyte ratio were the major negative regulating factors (Figure 6b). Altogether, we identified several early immune factors that can predict the magnitude of T-cell response and potentially predict vaccine efficacy.

### ZOSAL demonstrated a superior safety profile than Shingrix in rhesus macaques

**Figure 7.**
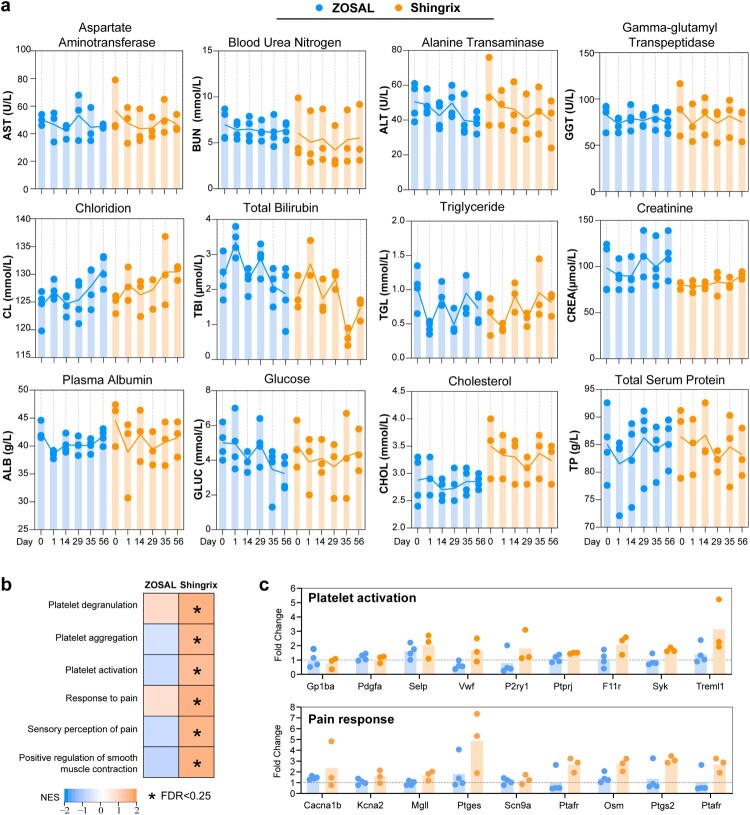
A preliminary evaluation of safety profiles of ZOSAL and Shingrix in rhesus macaques. Rhesus macaques were i.m. immunized twice with ZOSAL (n = 4) or Shingrix (n = 3) at an interval of 4 weeks. a. Levels of serum biochemical parameters at the indicated time points were measured. b. GSEA analysis revealed distinct differences in the level of indicated modules related to platelet function and pain response. Each box represents a specific module and colours indicate normalized enrichment score (NES). The asterisk denoted in the box represents False Discovery Rate (FDR) values < 0.25. c. Expression of indicated genes after vaccination. Data is shown as fold change normalized to pre-vaccination.

A promising vaccine candidate should possess a high safety profile. Despite the fact that Shingrix is highly effective in preventing zoster, clinical trials, and post-marketing surveillance have reported a high local and systemic reactogenicity of Shingrix [36]. In this study, we preliminarily evaluated the safety properties of ZOSAL by monitoring levels of various serum biochemical parameters following vaccination, including liver and kidney enzymes. In general, both ZOSAL and Shingrix-vaccinated macaques were well-tolerated and these parameters remained stable after vaccination (Figure 7a). Further, by revisiting the GSEA analyses on RNA-sequencing data, we noticed that modules associated with platelet degranulation, aggregation, activation, and pain responses were largely increased upon Shingrix vaccination, which in contrast were unchanged or even decreased after ZOSAL vaccination. Several genes that are typically relevant to platelet activation and pain response were further isolated and shown (Figure 7b, c). Collectively, our data indicated a high-level safety property of ZOSAL that is even superior to licensed vaccine.

## Discussion

Herpes zoster remains an important global health issue given the continuously rising incidence and limited licensed vaccines available [9]. Shingrix, an adjuvanted recombinant subunit vaccine, has a remarkably high protective efficacy and therefore has been dominating the market since its licensure. However, high reactogenicity that is likely caused by the strong AS01B adjuvant component remains a major concern on Shingrix [37,38]. There is therefore a critical need for novel or improved VZV vaccines with high efficacy and low reactogenicity.

VZV-specific T-cell response is solely critical for controlling latent reactivation and therefore must be considered in next-generation rational VZV vaccine development [12,13]. By now it is widely accepted that mRNA vaccines are potent at inducing T-cell responses. Both CD4^+^ and CD8^+^ T cell responses have been shown to be induced at higher levels by mRNA vaccines than induced by other protein vaccine platforms [39]. Therefore, for infections like VZV, the mRNA platform may be ideal to be employed for vaccine development. In the present study, we developed a novel VZV mRNA vaccine candidate (ZOSAL) and performed multiple assays to thoroughly evaluate vaccine immunogenicity and safety aspects with a side-by-side comparison with Shingrix. In adult mice, aged mice, and rhesus macaques, ZOSAL demonstrated superior immunogenicity and safety in multiple aspects over Shingrix, especially in the induction of strong T-cell immunity. Moreover, we took a step further to obtain mechanistic insights into the generation of vaccine-induced T-cell response and identified the associating immune correlates.

It has been reported that host immune responses to VZV infection involve both Ab and T cell responses [12,40]. While VZV-specific T-cell responses decline with age and their frequencies inversely correlate with the incidence or disease severity of HZ, the anti-VZV Abs largely remained stable or even increased with age and showed no association with HZ incidence or severity [41,42]. Moreover, an increased risk of HZ in immunocompromised individuals was associated with impaired T cell immunity but not with anti-VZV IgG titres [43]. Although these indicated that VZV-specific Abs are dispensable in protection against VZV, we still measured the Ab and B cell responses in our study, which we believe are critical indicators reflecting the phenotype and longevity of vaccine response. We found that ZOSAL elicited a comparable level of gE-specific IgG to Shingrix in mice, and the induced IgG subclass was much skewed towards a Th1-type IgG2c which suggested a more Th1-prone vaccine response induced by ZOSAL. Despite the distinct IgG subclass composition induced by the two vaccines, no clear difference in the effector functions of Abs was demonstrated. Previous studies have reported that Ab function is affected not only by the subclass but also by the patterns of glycosylation of Fc region [44–46], and distinct vaccine regimens induce different IgG glycosylation profiles [47]. It should be noted that ZOSAL and Shingrix represent two types of vaccines and their interactions with the immune system and the resulting immune activation profiles are quite different. Whether ZOSAL and Shingrix induce different glycosylation patterns of Abs and how this influences vaccine efficacy is an interesting topic to be further investigated. Neutralizing capacity of Abs was not assessed in our study due to technical reasons, and the exact role of neutralizing Abs in the control of VZV infection remains yet unclear. Regarding the B cell response, class-switched gE^+^ MBCs induced by ZOSAL were at a significantly higher level than that induced by Shingrix, which suggested ZOSAL was likely to induce a more sustained vaccine response than Shingrix, although not directly detected in our study.

T-cell immunity was particularly focused in our study due to the aforementioned reasons. In different animal models tested, ZOSAL demonstrated a significantly higher potency at eliciting VZV-specific Th1-type CD4^+^ T cell responses than Shingrix. However, we did not observe a clear induction of VZV-specific CD8^+^ T cells by the two vaccines. This was possibly due to the low frequency of VZV gE-specific CD8^+^ T cells or the low sensitivity of immune assays used. Moreover, the gE (ORF68) antigen may contain limited CD8^+^ T cell epitopes since it has been reported that VZV-specific CD8^+^ T cells were largely reactive to ORF9, not to other VZV antigens including gE and gB [48]. Another aspect that should be noted is that the VZV-specific T cells induced by ZOSAL did not show a dose-dependent pattern, which was in contrast to the induction of Abs. Similar findings have also been reported previously in many other mRNA vaccine studies [49–51]. So far, the mechanisms of action of mRNA vaccines remain largely unclear and await further in-depth investigation.

Effective generation of pathogen-specific T cells is largely determined by sufficient innate immune stimulation, especially the events associated with antigen presentation. Differences in the early innate responses induced by the two vaccines were in-depth characterized using non-human primate model. Both ZOSAL and Shingrix exhibit powerful abilities to activate innate immune compartments, in particular Type-I IFN signalling and antigen processing/presentation. Although we did not observe a striking difference between the two vaccine groups, this was quite expected since the adjuvant component in Shingrix that contains liposomes formulated with cholesterol, monophosphoryl lipid A (MPL), and QS21 was highly immune-stimulatory to favour the generation of Th1-type vaccine responses [52]. However, it should always be aware that innate activation is a “double-edged sword” and has to be well-controlled to an appropriate degree to aid in the generation of high-quality adaptive vaccine responses and at the same time ensure safety. Over-activated type-I IFN response has also been reported to be deleterious to the generation of Tfh response favouring Ab production [53]. This may unlikely occur to our mRNA vaccine since ZOSAL demonstrated superior ability in eliciting both class-switched gE^+^ MBCs and Tfh cells than Shingrix in mice.

In this study, we also performed a correlation analysis of 44 immune parameters to better understand the generation of vaccine-induced VZV immunity. It is easy to understand the strong positive correlations between the expression of IFN-relevant genes and the magnitude of T-cell responses. Interestingly, a few parameters including neutrophil/lymphocyte ratio at 24 h after vaccination, ADCP function of Abs, etc were negatively associated with T cell responses. Previously, we have reported that the number of circulating neutrophils increased rapidly and transiently 24 h after influenza or rabies mRNA vaccination in rhesus macaques [32,54]. Neutrophils are potent at phagocytosing LNP-mRNA particles but have a very limited capability to translate mRNA into the protein antigens [54]. This may cause an ineffective usage of injected mRNA vaccine that negatively affects the generation of vaccine responses.

We also took advantage of the rhesus macaque study to preliminarily assess the safety aspects of our VZV mRNA vaccine. A set of genes associated with platelet activation and pain responses were upregulated after Shingrix vaccination, but not by ZOSAL. Considering that aged individuals are the main target population of the zoster vaccine and these cohorts are more susceptible to cardiovascular diseases, the inappropriate platelet activation induced by Shingrix may pose a potential risk. Besides, the increased expression of pain-related genes also supported the clinical findings that local injection site pain is the most common solicited adverse event in Shingrix recipients [36]. In addition, it should be noted that our mRNA vaccine encodes full-length gE, which is in contrast with Shingrix that contains a truncated form of gE (1-544 aa) [55]. The rationale behind this design was mainly due to the fact that C-terminal domain of gE also contains efficient T cell epitopes that contribute to a broader induction of VZV-specific T cells [26]. However, further investigation would be necessary to address whether using full-length gE as a vaccine immunogen poses potential safety risks.

There are also some weaknesses of our study that remain to be further addressed. Dose titration of ZOSAL in rhesus macaques was not performed which was largely due to the limited source of animals and extraordinary high cost. However, this is critical to guide further studies on ZOSAL using a more optimal dose, especially in the potential clinical studies. In addition, we were not able to perform a viral challenge study to directly assess the protective efficacy of ZOSAL, although the superior T cell immunity induced by ZOSAL may suggest a superior protection. In fact, there are no adequate animal models of VZV infection that can fully recapitulate VZV reactivation in humans due to the strict host-specificity of VZV infection [56].

To summarize, we have developed a novel VZV mRNA vaccine candidate that has superior immunogenicity and safety property over licensed vaccine in mice and rhesus macaques. To our knowledge, this is the first study that provides high-resolution profiling and comparison of a VZV mRNA vaccine (ZOSAL) and Shingrix side-by-side in both mice and rhesus macaques. Importantly, our data generated from non-human primate model that is much appreciated for its high translational value for human vaccine responses will warrant more investigations of the mRNA platform in the development of next-generation herpes zoster vaccines.

## Supplementary Material

Huang_et_al_revised_Supplemental_Materials

## Data Availability

All data are available upon reasonable request to the corresponding authors.
